# Distinct EpCAM-Positive Stem Cell Niches Are Engaged in Chronic and Neoplastic Liver Diseases

**DOI:** 10.3389/fmed.2020.00479

**Published:** 2020-09-02

**Authors:** Samira Safarikia, Guido Carpino, Diletta Overi, Vincenzo Cardinale, Rosanna Venere, Antonio Franchitto, Paolo Onori, Domenico Alvaro, Eugenio Gaudio

**Affiliations:** ^1^Department of Precision and Translational Medicine, Sapienza University of Rome, Rome, Italy; ^2^Department of Movement, Human and Health Sciences, Division of Health Sciences, University of Rome “Foro Italico,” Rome, Italy; ^3^Department of Anatomical, Histological, Forensic Medicine and Orthopedic Sciences, Sapienza University of Rome, Rome, Italy; ^4^Department of Medico-Surgical Sciences and Biotechnologies, Sapienza University of Rome, Latina, Italy

**Keywords:** progenitor cells, liver, biliary tree, cholangiopathy, cholangiocarcinoma

## Abstract

In normal human livers, EpCAM^pos^ cells are mostly restricted in two distinct niches, which are (i) the bile ductules and (ii) the mucous glands present inside the wall of large intrahepatic bile ducts (the so-called peribiliary glands). These EpCAM^pos^ cell niches have been proven to harbor stem/progenitor cells with great importance in liver and biliary tree regeneration and in the pathophysiology of human diseases. The EpCAM^pos^ progenitor cells within bile ductules are engaged in driving regenerative processes in chronic diseases affecting hepatocytes or interlobular bile ducts. The EpCAM^pos^ population within peribiliary glands is activated when regenerative needs are finalized to repair large intra- or extra-hepatic bile ducts affected by chronic pathologies, including primary sclerosing cholangitis and ischemia-induced cholangiopathies after orthotopic liver transplantation. Finally, the presence of distinct EpCAM^pos^ cell populations may explain the histological and molecular heterogeneity characterizing cholangiocarcinoma, based on the concept of multiple candidate cells of origin. This review aimed to describe the precise anatomical distribution of EpCAM^pos^ populations within the liver and the biliary tree and to discuss their contribution in the pathophysiology of human liver diseases, as well as their potential role in regenerative medicine of the liver.

## Introduction

In the adult liver and biliary tree, mature parenchymal cells (i.e., hepatocytes and cholangiocytes) are characterized by remarkable proliferative capabilities, which support the regenerative needs of these organs in physiological conditions (1). According to that, lineage tracing studies indicated that hepatocytes are able to restore hepatic parenchyma after subcritical liver resection and regenerate cell loss after acute and chronic damage; furthermore, studies on cell plasticity in rodents indicated the potential of hepatocytes to differentiate toward biliary cells after injury (1–5). Therefore, conflicting evidence is present in scientific literature regarding the existence and function of a distinct stem/progenitor cell population capable of participating in liver regeneration (6). Nonetheless, experimental models characterized by an extensive (or prolonged over time) liver injury, which mimic the natural history of human liver diseases, demonstrated the contribution of stem/progenitor cells in the regenerative response (7, 8).

Recent single cell transcriptomic studies turned the spotlight on the EpCAM^pos^ cell fraction within the liver as a candidate progenitor cell population (9, 10). The epithelial cell adhesion molecule (EpCAM) is a surface epithelial marker selectively expressed in the biliary tree, where it individuates the stem/progenitor cell fraction from mature cholangiocytes (1, 11); differently, mature hepatocytes within the hepatic lobule do not express EpCAM (11). The elegant single-cell transcriptomic atlas compiled by Aizarani et al. produced a comprehensive depiction of liver cell populations, including hepatocytes, biliary epithelial cells, endothelial cells, macrophages, and inflammatory cells (9). The authors revealed the presence of transcriptomic heterogeneity in the EpCAM^pos^ population and, within this population, identified the cell fraction with the highest potential to form liver organoids and to putatively serve as a stem cell compartment. In parallel, the study by Segal et al. individuated, by using a similar approach, a unique gene expression profile within the EpCAM^pos^ cell population in human fetal livers: in particular, they individuated a distinct *EpCAM*^pos^ cell fraction which has bi-potential capability, is distinguishable from hepatocytes and cholangiocytes, and persists in the adult organ (10). Besides some discrepancies in cell phenotype, both studies individuated the *EpCAM*^pos^ population within the intrahepatic biliary tree as a strong candidate stem/progenitor cell compartment with regenerative potential for the liver and biliary tree.

The present review aimed to discuss the phenotype and the precise anatomical distribution of EpCAM^pos^ cell populations within the liver and the biliary tree, to describe their changes induced by human liver diseases, and to highlight their role as a potential source for regenerative medicine.

## The Heterogeneity of EpCAM^pos^ Cell Populations in Human Liver

**Figure 1:**
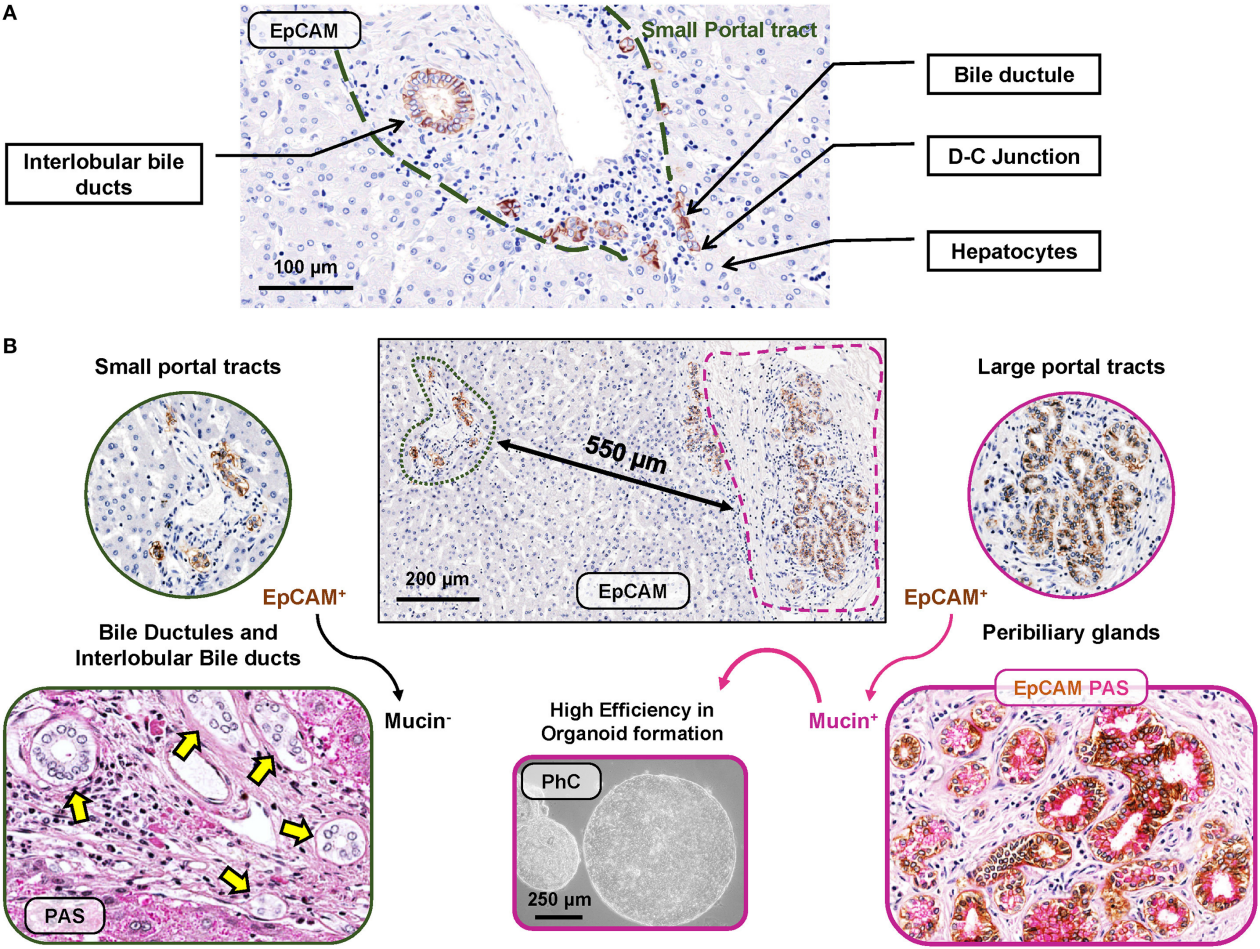
EpCAM^pos^ cell niches in human liver fragments obtained from normal subjects. **(A)** EpCAM^pos^ cells are present in small portal tracts and are endowed in interlobular bile ducts and bile ductules up to the ductular-canalicular (D-C) junction. Hepatocytes are constantly negative for EpCAM. **(B)** Large portal tracts can be present together with small ones in the same liver fragment, given their close proximity in sectional histology. Large intrahepatic bile ducts (i.e., segmental and area bile ducts) contain an EpCAM^pos^ cell niche endowed in the mucous glands present inside their walls (so-called peribiliary glands). Mucin positivity is typically present in EpCAM^pos^ peribiliary gland cells (on the right) but not in bile ductules and interlobular bile ducts at small portal tracts (on the left). Once isolates from peribiliary glands, EpCAM^pos^ cells showed progenitor cell features, including high efficiency in organoid formation *in vitro*. Histologic images are representatives of human liver fragments obtained from liver donors (*N* = 5). Specimens were stained by immunohistochemistry for EpCAM and periodic acid-Schiff (PAS) for mucins. EpCAM immunohistochemistry is counterstained with hematoxylin or with PAS. Organoids were generated by EpCAM^pos^ peribiliary gland cells isolated from the human common hepatic duct obtained from organ donors (routinely discarded in orthotopic liver transplantation procedures); the phase contrast (PhC) microscopic image is representative of at least *N* = 3 biological replicates.

In human livers, EpCAM^pos^ cells are localized and organized in two distinct anatomical niches (Figure 1), namely, bile ductules and peribiliary glands (PBGs).

**Figure 2:**
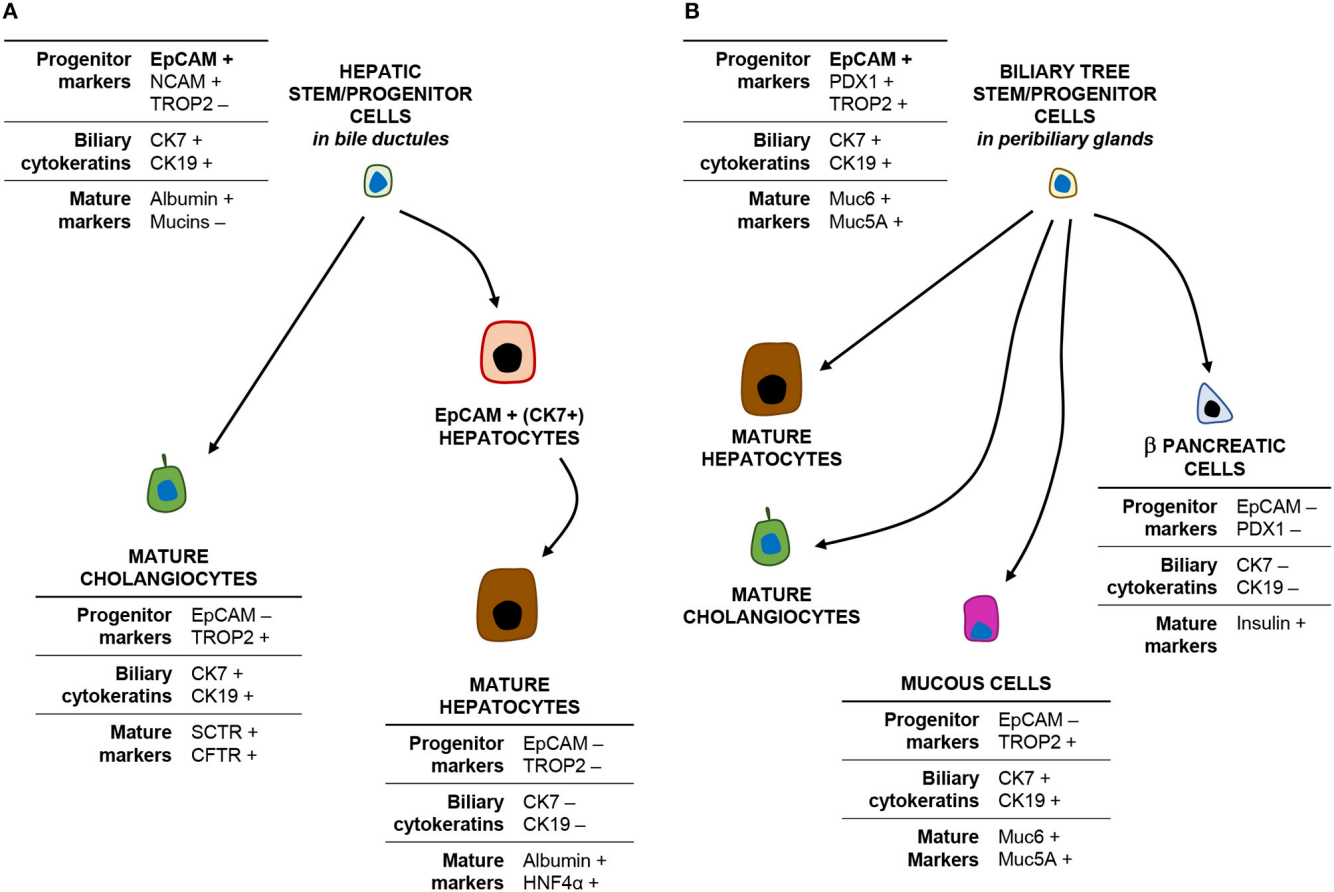
EpCAM^pos^ cells within the liver and biliary tree and their progeny. The cartoon shows the phenotype (main markers) of EpCAM^pos^ stem/progenitor cells within the bile ductules **(A)** and the peribiliary glands **(B)** and their differentiative capabilities.

The intrahepatic biliary tree begins with the canals of Hering, which represent the point of junction between the hepatocyte canalicular system and the biliary tree (12, 13). The canals of Hering are located at the interface between the portal tract and the hepatic parenchyma and continue into the bile ductules, with tortuous conduits draining into the interlobular bile ducts inside the portal space. A population of EpCAM^pos^ cells has been identified within the canals of Hering and the bile ductules, serving as facultative bipotent progenitors (Hepatic Stem/progenitor Cells: HpSCs) capable to differentiate into hepatocytes and cholangiocytes (Figure 2, panel A) (9, 14, 15), and represents the remnant of the ductal plate in the adult liver (10, 16, 17). Morphologically, HpSCs are small cells characterized by a high nucleus-to-cytoplasm ratio and expressing a large variety of markers, which include stem cell markers [e.g., EpCAM, neural cell adhesion molecule (NCAM), transcription factor Sox9, CD44, and CD133], biliary cytokeratins (CK7/19), and hepatocellular traits (e.g., albumin, CK18, hepatocyte nuclear factor 4 alpha) (10, 18). While differentiating toward a mature fate, the progeny of HpSCs is characterized by the progressive loss of EpCAM and NCAM expression and the acquirement of mature hepatocyte or cholangiocyte traits (10, 14, 19, 20). Recently, this EpCAM^pos^ cellular population has been further characterized by single-cell transcriptomic, with the identification of an *EpCAM*^po*s*^*/NCAM*^pos^ fraction which also displays prominent stem cell features *in vitro* (10).

Notably, a second EpCAM^pos^ cell niche is endowed in PBGs located inside the walls of large (i.e., segmental and area) intrahepatic bile ducts and along the entire extrahepatic biliary tree (21). PBGs are tubulo-alveolar mucous glands, in continuity with the surface epithelium of the bile duct (22, 23). Intriguingly, the EpCAM^pos^ cell population within PBGs showed stem/progenitor properties, including organoid formation and plasticity to differentiate into hepatocytes, cholangiocytes, and endocrine pancreatic cells (Figure 2, panel B) (21, 24, 25). PBG cells have been collectively named biliary tree stem/progenitor cells (BTSC) and, embryologically, they represent the remnant of the common bilio-pancreatic progenitors of the ventral endoderm (23, 24). Phenotypically, mucin family genes (e.g., *MUC5A* and *MUC6*) are largely expressed in this anatomical niche (23); moreover, the EpCAM^pos^ cell pool within PBGs shows a heterogeneous profile and a radial axis (depth-to-surface) organization: EpCAM^pos^ cells are mostly found at the bottom of PBGs, where a subpopulation of them co-expresses markers of pluripotency (i.e., Oct4, Sox2, and Nanog) (23); a transit-amplifying (i.e., proliferating) population is located in the middle portion of the glands; finally, cells with a more mature phenotype [e.g., expressing secretin receptor, cystic fibrosis transmembrane conductance receptor (CFTR), mucins, and Trop2] are located in direct continuity with the surface epithelium (23, 26, 27). Altogether, these findings are confirmed by single-cell transcriptomic analysis in human, with the identification of an *EpCAM*^pos^/*TROP2*^int^/*MUC6*^high^ progenitor compartment with prominent stem cell features (9). Moreover, the contribution of PBG cells in the regeneration of mature biliary epithelium has been demonstrated in experimental conditions and by an *ex vivo* human model of biliary regeneration, which have disclosed that PBG cells can repopulate the surface epithelium of bile ducts by proliferation and differentiation into mature cholangiocytes (25, 28, 29).

In the light of these findings, one of the main aspects to be considered when interpreting transcriptomic analysis of liver samples resides in the fact that PBGs and bile ductules are two anatomically distinct compartments which, however, can be found in strict spatial proximity within the same liver fragment (Figure 1, panel B). Thus, the transcriptomic heterogeneity in the EpCAM^pos^ population revealed by single cell approaches could be due to the collection of one or both of these distinct progenitor cell compartments from the same specimen. EpCAM^pos^ progenitor cells within bile ductules are characterized by a distinct signature identifying them from mature cholangiocytes, immature hepatocytes, and mature hepatocytes, based on *NCAM*^pos^/*TROP2*^neg^ expression (Figure 2). Therefore, these cells could be distinguished from cholangiocytes that populate interlobular bile ducts, the latter expressing *TROP*2 and mucin family genes. However, a *TROP*^int^ cell fraction among the EpCAM^pos^ compartment was proven to have high stemness properties; thus, this fraction resides more likely into peribiliary glands than in bile ductules, given also its high mucin family gene expression (e.g., *MUC5A* and *MUC6)* and low CK19 and CFTR levels (25, 30).

Understanding the phenotypes of distinct biliary populations and their anatomical niches is crucial when considering that the pathophysiology of hepatic and biliary diseases is often hindered by difficulties in identifying regenerative pathways. Interestingly, the EpCAM^pos^ population exhibited only a stochastic expression of proliferation markers (9). This finding strengthens the concept of a facultative progenitor compartment that is only engaged by the regenerative needs and trajectories required after prolonged chronic or acute massive damage (8).

## EpCAM^pos^ Cell Populations in Chronic Human Diseases of the Liver and Biliary Tree

**Figure 3:**
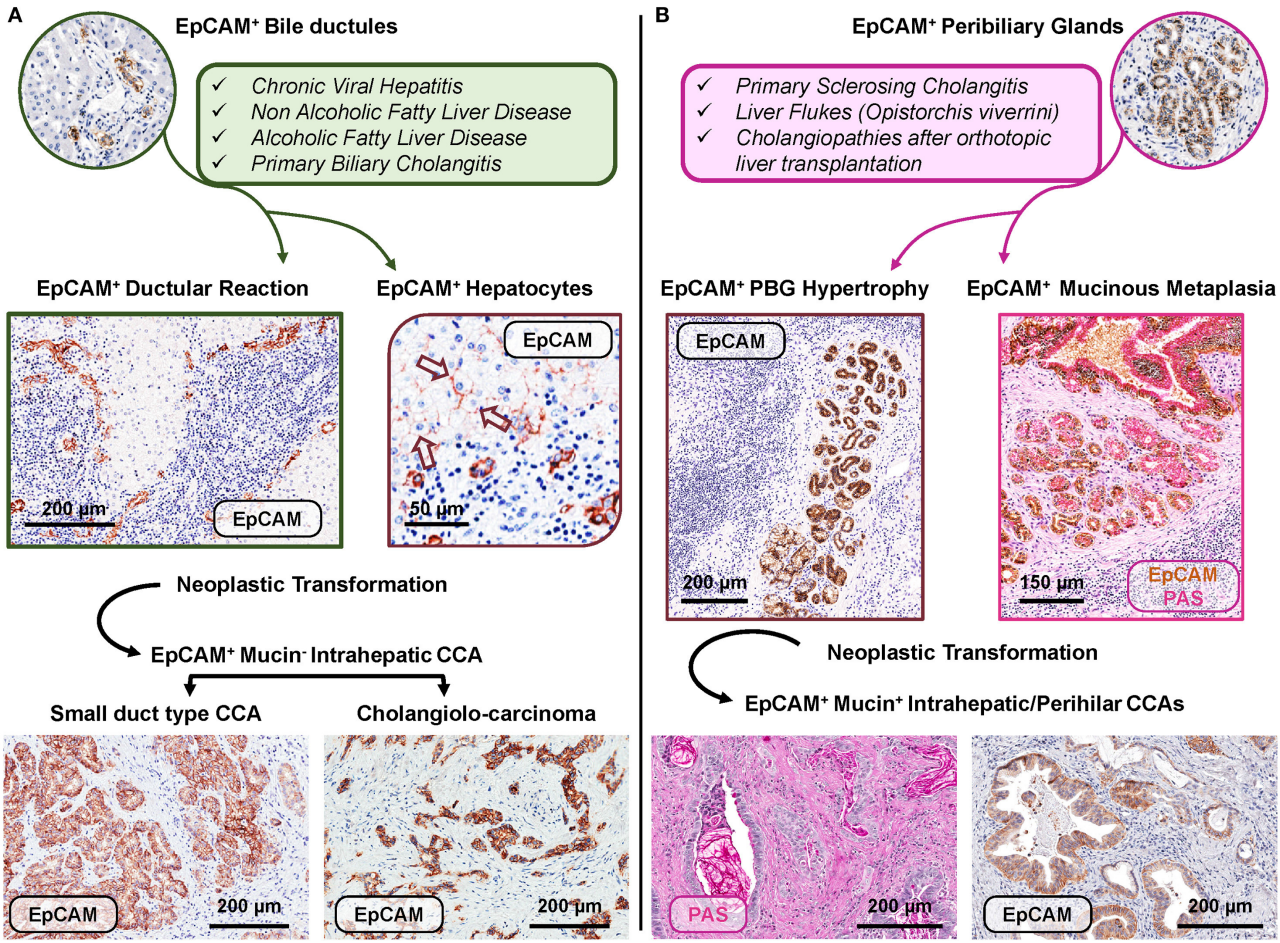
Distinct EpCAM^pos^ cell niches are engaged by specific human diseases based on regenerative needs and may represent the cell of origin of specific subtypes of cholangiocarcinoma. **(A)** Regenerative pathways engage EpCAM^pos^/MUC6^neg^ progenitors within bile ductules in chronic human diseases affecting hepatocytes and/or interlobular bile ducts. In these diseases, an expansion of the EpCAM^pos^ cell population is present and includes the emergence of ductular reaction and the appearance of EpCAM^pos^ hepatocytes (arrows). Two subtypes of EpCAM^pos^ cholangiocarcinoma (CCA) may derive from the neoplastic transformation of EpCAM^pos^ interlobular cholangiocytes (small duct type CCA) and bile ductules (cholangiolo-carcinoma). **(B)** The EpCAM^pos^/MUC6^pos^ population within peribiliary glands (PBGs) could be engaged when regenerative needs are finalized to repair large intra- or extrahepatic bile ducts affected by chronic pathologies, including primary sclerosing cholangitis. The EpCAM^pos^ PBG cell population response is characterized by hypertrophy and mucinous metaplasia of this compartment. The onset of mucinous CCAs could be due to the neoplastic transformation of the EpCAM^pos^ PBG niche, through the sequence hypertrophy-metaplasia-dysplasia-cancer. Histologic images in **(A)** are representative of human liver fragments obtained from patients affected by chronic viral hepatitis, primary biliary cholangitis, and CCA. In **(B)**, specimens were obtained from patients affected by primary sclerosing cholangitis and CCA. Specimens were stained by immunohistochemistry for EpCAM and periodic acid-Schiff (PAS) for mucins. EpCAM immunohistochemistry is counterstained with hematoxylin or with PAS.

In human liver diseases, the phenotypic and transcriptomic profiles of liver cells change noticeably. In this context, taking into account the heterogeneity within the EpCAM^pos^ progenitor population is a crucial prerequisite to understand the specific contribution of different hepatic regenerative pathways in human disease progression. Chronic prolonged diseases affecting hepatocytes or interlobular bile ducts could engage HpSCs within bile ductules (Figure 3, panel A) (19); differently, PBGs and their EpCAM^pos^ cell fraction (i.e., BTSCs) could be activated when the disease leads to the damage of large intrahepatic (or extra-hepatic) bile ducts (Figure 3, panel B) (30, 31).

### EpCAM^pos^ Cell Fraction in Bile Ductules: Ductular Reaction and Chronic Liver Diseases

In a number of different chronic liver diseases, EpCAM^pos^ HpSCs within the canals of Hering and the bile ductules represent the source of a prominent ductular reaction (DR) (Figure 3, panel A) (19, 32). The phenotype and the behavior of EpCAM^pos^ DR have been extensively characterized in liver diseases affecting hepatocytes (19). In these conditions, DR appearance is associated with the emergence of a unique EpCAM^pos^ hepatocyte population, which localizes periportally and is characterized by the co-expression of biliary cytokeratins (11, 19, 33). Detailed studies of liver samples obtained from cirrhotic patients allowed accurately describing the expansion of DR and the emergence of an EpCAM^pos^ hepatocyte population, able to regenerate large portions of liver parenchyma (34, 35).

In chronic liver diseases, the expansion of DR is secondary to the prolonged exposure of hepatocytes to pathogenetic insults, which leads to the progressive loss of hepatocyte replicative capability and induces hepatocellular senescence. For example, in non-alcoholic fatty liver disease (NAFLD), hepatocyte regenerative capabilities are highly impaired due to progressive lipotoxicity and lobular inflammation (36, 37); this induces a prominent EpCAM^pos^ DR, followed by the appearance of EpCAM^pos^ hepatocytes at the periportal zone (37–42). In NAFLD, DR correlates with disease activity and NASH onset; interestingly, DR extent also correlates with periportal fibrogenesis and is predictive of fibrosis stage (43, 44). Parallel studies confirmed this role of DR also in pediatric subjects affected by NASH (39, 45). Moreover, DR is associated with systemic oxidative stress levels (38) and extra-hepatic clinical manifestations such as obstructive sleep apnea syndrome (46).

Extensive EpCAM^pos^ DR can also be triggered by a severe hepatocyte loss as a consequence of acute liver injury. In alcoholic hepatitis, a relevant cause of morbidity and mortality in heavy drinkers with alcoholic liver disease (47), DR extension is correlated with the severity of the damage and can predict short-term mortality (47, 48). In this setting, patients responding to first-line steroid therapy were characterized by higher DR (49); differently, in non-responders, cells within DR do not show signs of differentiation into hepatocytes, which correlates with a less favorable outcome (19, 50–53).

A prominent EpCAM^pos^ DR also appears in chronic diseases affecting the biliary tree (i.e., cholangiopathies) and acquires a peculiar phenotype, which reflects the pathophysiology of the disease. Primary biliary cholangitis (PBC) is a chronic, autoimmune cholangiopathy, characterized by the damage of interlobular bile ducts, leading to impaired bile duct flow into the biliary tree (54). In patients affected by PBC, DR is composed of cells expressing stem cell markers (EpCAM, NCAM, Sox9, and CD133) and displaying a variable degree of cholangiocyte differentiation signs, together with Notch pathway activation (19, 33, 55, 56). Disease severity and stage are correlated with the extent of DR, which might serve as prognostic marker for the development of clinical symptoms, patient prognosis, and response to therapy (55, 57). Differently from PBC, patients affected by primary sclerosing cholangitis (PSC) show a unique DR phenotype. In PSC, chronic inflammatory damage targets large intrahepatic bile ducts and mostly spares interlobular bile ducts; the development of fibrotic strictures and consequent cholestatic injury of the liver parenchyma trigger DR which, however, is less prominent compared to PBC and is characterized by the expression of hepatocellular traits, with the appearance of numerous EpCAM^pos^ hepatocytes (55).

In summary, liver diseases induce a substantial modification of the EpCAM^pos^ fraction within the liver; these changes are strongly influenced by the etiology of liver diseases and the specific regenerative needs. Remarkably, liver diseases further re-shape the non-parenchymal cell fractions (i.e., macrophages and hepatic stellate cells), inducing an inflammatory and fibrogenetic response (Table 1) (37, 40). In turn, these changes influence the EpCAM^pos^ cell compartment and support the vicious cycle which promotes and sustains liver fibrosis (58, 59). This represents the rationale for the strong correlation between EpCAM^pos^ fraction activation and clinical outcomes in human diseases (40, 57).

**Table 1:** Summary of the main signaling pathways involved in EpCAM^pos^ niches of the liver and biliary tree.

**Pathway**	**Secreted ligand**	**Receptor**	**Intracellular effector**	**Function**
Notch	Jagged 1 Source: HSCs/MFs	Notch	Notch intra-cellular domain	Cholangiocyte fate
Wnt/β-catenin	Wnt1/Wnt3a Source: macrophages	P-β-Catenin	β-Catenin	Proliferation Hepatocyte fate
Hedgehog	Hh Source: EpCAM^pos^ stem/progenitor cells	Patched	Gli-1	Fibrogenesis EMT
Wnt/planar cell polarity	Wnt5a Source: HSCs/MFs	VANGL2	JNK/JUN	Modulation of niche microenvironment

*EMT, epithelial/mesenchymal transition; HSC, hepatic stellate cell; MF, myofibroblast*.

### EpCAM^pos^ Cells Within PBGs in Human Cholangiopathies

The EpCAM^pos^ cell population in PBGs is implicated in human diseases affecting large intrahepatic and extrahepatic bile ducts (Figure 3, panel B) (1). Indeed, in PSC samples, progressive hyperplasia, and mucinous metaplasia of PBGs characterize fibrotic large bile ducts. Hyperplasia of PBGs is determined by the expansion of BTSCs, which also contributes to biliary fibrosis trough epithelial-to-mesenchymal transition and is sustained by the signaling pathway mediated by hedgehog ligands (Table 1) (30, 55). Interestingly, in PSC patients, the expansion of PBG mass correlated with the severity of the histological stage of liver cirrhosis and with the clinical stages according the Mayo score (30). It is worth noting that the histopathology of large bile duct fibrosis (which is at the basis of biliary strictures development in PSC) was understudied for years, although a consensus on PSC animal models highlighted the need to study large bile ducts and associated PBGs (60). In PSC and in pathologies of large bile ducts and in extrahepatic cholestasis, indeed, inflammation mostly spares the interlobular bile ducts within the liver parenchyma, whereas ductular reaction and fibrosis are present (55). These observations indicate that parenchymal injury in PSC is the consequence of ascending cholestasis. Differently, in PBC patients, the histomorphological study of bile ducts and livers demonstrated no activation of the PBG niche, thus indicating that the EpCAM^pos^ BTSCs endowed in large intrahepatic and extrahepatic bile ducts are elicited by the injury of this portion of the biliary tree (as in PSC) but not in diseases affecting occurring interlobular bile ducts (i.e., PBC) (30).

Other than PSC, PBGs are involved in the turnover and regeneration of biliary epithelia in sclerosing reactions in secondary sclerosing cholangitis and hepatolithiasis (22, 61). Cystic changes in PBGs can occur *de novo*, as part of a congenital syndrome, or secondarily to insults such as alcoholic cirrhosis (61). Interestingly, the systematic examination of bile ducts of liver grafts revealed the relationship between the degree of ischemic-based PBG injuries and the future development of non-anastomotic strictures (28, 62). Moreover, using an *ex vivo* model based on precision-cut slices of extrahepatic human bile ducts obtained from discarded donor livers, the spatiotemporal differentiation and migration of PBG cells after severe biliary injury was recently studied; this approach revealed that human PBGs contain biliary progenitor cells and are able to respond to bile duct epithelial loss with proliferation, differentiation, and maturation to restore epithelial integrity, providing evidence for a pivotal role of PBGs in biliary regeneration after severe injury (28).

Although normal PBGs themselves are particularly small structures that cannot be recognized using any of the currently available imaging modalities, these glands are closely associated with several diseases which have typical imaging features. The knowledge of the basic pathophysiology of PBGs could be helpful for depicting innovative diagnostic modalities and endpoints in biliary diseases associated with PBG injury (61). Since human BTSC activation in fibrotic large duct pathologies is associated with PBGs hyperplasia/metaplasia and dysplasia, in the future, histomorphology and radiology correlation studies are needed in order to envision innovative PBG imaging tools.

Mechanisms underlying the repair of extrahepatic biliary tree after injury have been scarcely explored. We have recently shown that the Wnt signaling pathway triggers human BTSC proliferation *in vitro* and influences PBG hyperplasia *in vivo* in a mouse biliary injury model (25). In particular, Notch signaling pathway activation induces BTSC differentiation *in vitro* toward mature cholangiocytes, in parallel with the observation of Notch pathway activation within PBGs in a mouse model; moreover, in human PSC, inflammatory and stromal cells trigger PBG activation through the upregulation of the Wnt and Notch signaling pathways (25). Considering these findings, the demonstration of the involvement of PBG cells in regenerating the injured biliary epithelium and identifying the signaling pathways driving BTSC activation could have relevant implications on the treatment of cholangiopathies.

The EpCAM^pos^ (MUC6^high^) cell population may even respond to systemic stimuli, like hyperglycemia. In experimental and human diabetes, EpCAM^pos^/MUC6^high^ cell population in PBGs respond to diabetes with proliferation and differentiation toward insulin-producing cells, indicating that PBG niches may rescue pancreatic islet impairment in diabetes (63). These findings offer important implications for the pathophysiology and complications of this disease, comprising the understanding of the paramount role of type II diabetes in cholangiocarcinoma risk nowadays (see the next paragraph) (63).

## EpCAM^pos^ Cell Populations in Cholangiocarcinoma

The recent focus on the EpCAM^pos^/MUC6^high^ cell population residing in large intrahepatic and extrahepatic bile ducts sheds light on the clinical management of malignancies arising in this organ, which are collectively termed cholangiocarcinoma (30, 31). From an anatomical point of view, cholangiocarcinoma can be classified as intrahepatic, perihilar, or distal (64); however, growing evidence suggests that distinct cells of origin within an organ, particularly tissue-specific stem cells, may give rise to different cancer subtypes (Figure 3) (65). In this context, certain subtypes of intrahepatic cholangiocarcinoma (small bile duct type and cholangiolo-carcinoma) are constituted by mucin^neg^ cells and could originate from small cholangiocytes or from the EpCAM^pos^/MUC6^neg^ population located in ductules and/or interlobular bile ducts. In keeping, chronic liver diseases and cirrhosis represent specific risk factors for these tumor subtypes, and the surrounding parenchyma is generally characterized by a marked EpCAM^pos^/mucin^neg^ DR (40, 57). The division rate of stem cells is linearly correlated with the lifetime risk of developing primary liver cancer (66).

Intriguingly, mucinous intrahepatic (large bile duct type) and perihilar cholangiocarcinoma show a similar phenotype, with mucin^pos^ cells occupying the entire neoplastic mass, and are characterized by a similar clinical course (67); such tumors could arise from PBGs and, likely, from the EpCAM^pos^/TROP2^int^/MUC6^high^ compartment (31). PSC and liver flukes represent well-recognized risk factors for these tumor subtypes (67); these diseases chronically affect large intrahepatic and extrahepatic bile ducts and determine hyperplasia, mucinous metaplasia, and dysplasia in PBGs (30). The expansion of the EpCAM^pos^/MUC6^high^ compartment within PBGs represents an attempt to restore epithelial integrity as a response to chronic bile duct damage (25). However, the continuous exposure to harmful stimuli leads to a progressively uncontrolled response, and dysplasia-to-cancer progression takes place diffusely within bile ducts with multiple neoplastic foci, mimicking field cancerization (31).

The inter-tumor heterogeneity of cholangiocarcinoma might be due to the interplay of distinct tissues/cells of origin, the underlying disease, and the associated molecular clustering based on driver mutations which shape the pathobiological features of the different cholangiocarcinoma subtypes. In keeping with that, intrahepatic cholangiocarcinoma can be distinguished into two main subtypes (small and large bile duct type) based on its origin and histomorphologic features. Remarkably, these tumor subtypes have distinct cancer stem cell profiles (68), thus suggesting a putative different cell of origin (HpSCs vs. BTSCs). These subtypes also differ in terms of gross growth pattern, genetic signatures, and driver mutations (e.g., IDH1/2 in small bile duct type and KRAS in large bile duct type), and prognosis. With this latter regard, the large bile duct cholangiocarcinoma subtype is a highly aggressive cancer, characterized by a low disease-free and overall survival. This tumor typically arises in patients with PSC and has been proved to derive from dysplastic EpCAM^pos^ PBGs both in humans (31) and in experimental models (69).

Emphasizing the role of the different tissues/cells of origin on the complex pathogenesis of cholangiocarcinoma, also in the picture of EpCAM^pos^ cell niches, could have implications on preventive strategies and early diagnosis in patients with underlying clinical or subclinical hepatobiliary disease, shedding new light on dissecting cholangiocarcinoma heterogeneity and allowing a rational approach to personalized medicine for this devastating cancer (67, 70).

## Perspectives and Applications of EpCAM^pos^ Cells in Regenerative Medicine

Orthotopic liver transplantation represents the main curative option for the majority of chronic liver diseases. In this context, the shortage of organ donors represents a severe limitation to patients' treatment, which results in many patients dying while waiting for transplantation. For these patients, the development of effective strategies for cell therapy could represent a promising approach to the treatment of liver diseases, also as a bridge to liver transplantation.

Therefore, different strategies of cell therapy have been attempted (71, 72). Hepatocyte transplantation represents the proof of concept of liver cell therapy but it is limited by the scarcity of donor organs, the low cell engraftment, difficulties in cryopreservation, and the necessity of long-term immunosuppression (72–74). Mesenchymal-derived stem cells, comprising hematopoietic stem cells and mesenchymal stem cells, have been largely applied for liver regenerative medicine purposes in a number of clinical trials throughout the world (71, 75–79). Although autologous transplantation of hematopoietic and mesenchymal stem cells is clinically safe, the recent negative results of randomized clinical trials limit the future application of this strategy (75–79). Clinical applications of pluripotent stem cells in hepatology are actually null; this is due to the teratocarcinogenic risk and the ethical concerns for the use of embryonic stem cells and to the possibility of tumorigenic expansion of reprogrammed induced pluripotent stem cells (72).

In this scenario, the hepatic EpCAM^pos^ cell fractions could represent an important alternative source, currently under investigation worldwide. Other than case reports concerning the use of EpCAM^pos^ HpSCs in biliary atresia and inborn errors of metabolism (80, 81), a non-randomized clinical trial of 25 subjects and 25 controls with decompensated liver cirrhosis due to various causes undergoing human fetal EpCAM^pos^ HpSCs infusion into the liver via the hepatic artery has been reported (82). At 6-months follow-up, multiple diagnostic and biochemical parameters showed improvement, with a significant decrease in the patients' MELD (model for end-stage liver disease) scores (82). In western countries, Pietrosi et al. performed an intrasplenic infusion of a total cell population obtained from the fetal liver in nine patients affected by end-stage liver disease, demonstrating that the procedure was safe and well-tolerated in all patients (83). Similar to HpSCs, EpCAM^pos^ BTSCs could represent a possible source for stem/progenitor cells for liver regenerative medicine, as they are readily available from the biliary tree of adult or fetal organ donors and from cholecystectomized patients (84–87). In the past, we reported a preliminary experience in treating patients with advanced cirrhosis by the infusion via hepatic artery of human EpCAM^pos^ BTSCs (84). This report represents the proof of concept that EpCAM^pos^ BTSCs are a suitable source for the cell therapy of liver cirrhosis and represents the basis for an ongoing controlled clinical trial (84). Moreover, cells are routinely isolated in good manufacturing practices (GMP) conditions, and all required media are available in GMP-grade (84). Finally, the EpCAM-based sorting procedure is highly standardized and has been already used in clinical program (11, 84, 88, 89).

3D organoids represent an advanced culture technology in the field of stem cells and regenerative medicine, recapitulating embryonic development and the physiology of the tissue of origin. So far, organoid cultures have been developed for the adult intestine (90), stomach (91), and pancreas (92) of different species, like mouse and human. When cultured as organoids, cells can expand long term in culture, maintain the genetic stability, and spontaneously self-organize into structures that resemble the *in vivo* tissue in terms of cellular composition and function.

Organoid techniques have been largely applied to human liver, in particular to its EpCAM^pos^ stem/progenitor compartments. Under defined culture conditions, EpCAM^pos^ HpSCs from adult liver can be expanded for months (>1 year) in culture, showing an exponential rate of proliferation and genetic stability (92). HpSCs can readily be expanded as bipotent stem cells into 3D organoids and differentiate into functional hepatocyte cells both *in vitro* and *in vivo* upon transplantation (93, 94). Noteworthily, single isolated EpCAM^pos^ cells develop into organoid with high colony forming efficiency, while EpCAM^neg^ cells failed to create organoids (93). Moreover, the engraftment and differentiation of organoids was demonstrated in mouse models of acute liver injury; in these mice, a stable expression of human hepatocyte markers was observed in recipient serum for more than 4 months, and cell engraftment was confirmed by immunohistochemistry for human albumin (93).

The application of the organoid technique is feasible to obtain long-term expansion of BTSCs, maintaining stem cell markers and genetic stability over months (24, 87, 95). Interestingly, Sampaziotis et al. have demonstrated that cells isolated from the extrahepatic biliary tree can be expanded *in vitro* in long-term 3D organoid culture, while maintaining the biliary transcriptional signature and functional characteristics; this culture can be propagated as a potential system for the application of regenerative medicine in the field of cholangiopathies and *in vitro* common bile duct disease modeling (96).

Overall, organoid cultures derived from patients' liver could represent a relevant system to study human biology, physiology, and the regeneration and development of the liver and biliary tree (97). In addition, 3D organoid cultures enable the study of molecular mechanism driving liver diseases, comprising cholangiopathies (98) and primary liver cancers including cholangiocarcinoma (99); at the same time, this technique can provide potential tools to manipulating genomes and facilitate gene correction for regenerative medicine and autologous therapy (97). Recently, a specific interest has increased for the genetic modification of liver organoids to create disease-specific models in settings where providing a patient's material is challenging (100).

Liver organoids facilitate the study of human liver diseases and regeneration and open the way to expand human liver cells *in vitro* in the field of personalized medicine or drug testing for biliary and liver disease. The effective application of organoid-derived hepatocytes and cholangiocytes to humans needs to face important technical issues related to the *in vitro* manipulation and the large use of non-clinical-grade GMP substances necessary to promote and sustain long-term 3D growth.

## Conclusion

The liver and the biliary tree harbor two EpCAM^pos^ stem/progenitor cell niches, both yielding high regenerative capabilities and the potential to differentiate into mature hepatocytes and cholangiocytes, but displaying distinct localization and phenotype and peculiar behavior and role in the physio-pathogenesis of liver and biliary diseases.

EpCAM^pos^ HpSCs are located at the canals of Hering and in the bile ductules; these cells are recruited in human acute and chronic liver diseases affecting hepatocytes, contributing to liver regeneration and being implicated in the fibrogenetic processes. HpSCs are also elicited in cholangiopathies affecting interlobular bile ducts (i.e., primary biliary cholangitis). EpCAM^pos^ BTSCs can be found in the PBGs of large intrahepatic or extrahepatic bile ducts and participate in regenerative responses following cholangiopathies affecting large ducts. Their activation is key in the pathogenesis of biliary strictures in primary sclerosing cholangitis, and their injury is implicated in the onset of non-anastomotic strictures following orthotropic liver transplantation.

Both niches could contribute to the development of cholangiocarcinoma due to their prominent activation occurring in pre-neoplastic conditions, and their distinct phenotype could explain, at least in part, the heterogeneity observed in cholangiocarcinoma subtypes.

Finally, due to their stem cell properties and safety of use, they represent a valid cell source for regenerative medicine of the liver.

## Author Contributions

SS: study concept, design, and drafting of the manuscript. GC: study concept, design, drafting of the manuscript, figure preparation, and study supervision. DO, AF, and VC acquisition of images, figure preparation, drafting of the manuscript, and critical revision of the manuscript for important intellectual content. PO, DA, and EG critical revision of the manuscript for important intellectual content, obtained funding, and study supervision. All authors contributed to the article and approved the submitted version.

## Conflict of Interest

The authors declare that the research was conducted in the absence of any commercial or financial relationships that could be construed as a potential conflict of interest.

## Abbreviations

BTSC: biliary tree stem/progenitor cell
CD: cluster differentiation
CFTR: cystic fibrosis transmembrane conductance receptor
CK: cytokeratin
DR: ductular reaction
EpCAM: epithelial cell adhesion molecule
GMP: good manufacturing practice
HpSC: hepatic stem/progenitor cell
MUC: mucin
NAFLD: non-alcoholic fatty liver disease
NCAM: neural cell adhesion molecule
PBC: primary biliary cholangitis
PBG: peribiliary gland
PSC: primary sclerosing cholangitis.
